# Drugs for Diabetes: From Pharmacology to Clinical Application

**DOI:** 10.3390/ph16101346

**Published:** 2023-09-24

**Authors:** Swayam Prakash Srivastava

**Affiliations:** Hartman Institute of Therapeutic Organ Regeneration, Division of Regenerative Medicine, Department of Medicine, Weill Cornell Medicine, New York, NY 10065, USA; swayam.cdri@gmail.com or sps4004@med.cornell.edu

Type I and type II diabetes mellitus, characterized by increased blood glucose levels, affect almost half a billion people around the world. Diabetes is caused either by an inability to produce enough insulin or insufficient insulin action. Regardless of diabetes type, its complications involve microvascular, macrovascular, and neuropathic issues [1]. Microvascular and macrovascular complications include nephropathy, retinopathy, neuropathy, cardiovascular disease, dyslipidemia, and hypertension [1]. Diabetes mellitus accelerates mesenchymal activations in organs such as the kidneys and heart, influencing the pathways that regulate extracellular matrix (ECM) synthesis [2]. The management of diabetes mellitus without any side effects is a challenge to the medical system. Very few specific therapeutics exist that minimize diabetic risk and mitigate its complications. In a preclinical setting, regulating oxidative stress, improving the quality of mitochondria, and targeting the pathways in diabetic complications have shown encouraging outcomes [3]. Such interventions present a new approach for the management of diabetic complications, but further investigations are needed for better management.

Several molecules, such as flavones, isoflavone, and chalcones, have shown promising activity as inhibitors against drug targets, i.e., PTP1B, α-glucosidase, DPP-4, aldose reductase, SGLT-2, etc., in type 2 diabetes [3,4,5]. These identified molecules have better efficacy in mouse models of diabetes mellitus when compared to standard drugs. In recent years, molecules targeting tissue-specific ANGPTL4, MST-1 inhibitors, and SGLT-2 inhibitors have been shown to be effective in combating diabetes mellitus. Moreover, these data suggest that catechol-o-methyl transferase (COMT) deficiency can lead to metabolic abnormalities such as diabetes mellitus, gestational diabetes, and pre-eclampsia in mice [6]. Treatment with the COMT by-product 2-methoxy estradiol traversed the phenotype of metabolic syndrome in the mice; however, small- and large-scale random clinical trials are needed in patients before developing 2-ME as a medicine for human use [6]. The function of antidiabetic medications, with known thermogenic mechanisms that are involved in adipose-tissue-mediated thermogenesis, can be targeted to combat obesity and related diabetes [7]. Such mechanisms play a crucial role in bettering our understanding of non-shivering thermogenesis and in the development of new therapeutic medications for diabetes and its related complications.

This Special Issue covers new pathways and mechanisms in diabetes mellitus, investigating new approaches for the management of organ fibrosis in diabetes. In addition, the Special Issue helps to identify new management strategies for the disease using new therapeutic approaches.

We broadly focused on two major sections.

## 1. New Molecular Mechanisms in Diabetes

Endothelial cells function by maintaining vascular integrity, homeostasis, and barrier function, and by arresting inflammation through regulating their interactions with immune cells. Endothelial cell dysfunction is the predominant pathology of pre-thrombotic complications in diabetes. Endothelial-to-mesenchymal transition (EndMT) is one of the mechanisms by which endothelial cells acquire the characteristics of mesenchymal phenotypes [8]. Alterations in endothelial cell polarity and EndMT activation are the key phenomena that accelerate fibrogenic pathways, resulting in the accumulation of an extracellular matrix and fibrosis-related proteins [9,10]. My research is predominantly focused on the identification of key endogenous molecules that are linked to endothelial cell homeostasis and these are: (1) endothelial glucocorticoid receptors (GR) nuclear receptors and their deficiency, which causes the activation of Wnt-associated mesenchymal activations and a linked disruption in fatty acid metabolism in the endothelial cells themselves, and also in neighboring cells, resulting in severe fibrogenic responses in diabetic kidneys and hearts [11,12,13]; (2) endothelial fibroblast growth factor receptor 1 (FGFR1), a cell surface receptor, a deficiency of which causes the activation of the mesenchymal mechanisms through downregulating the gene-expression level of antifibrotic microRNAs [14,15,16]; and (3) endothelial sirtuin 3 (SIRT3), a mitochondrial protein that regulates metabolic flux through targeting pyruvate kinase M2 tetramer-to-dimer formation [17,18] and the accumulative effects of these pathological features leads to endothelial cell leakage, alterations in endothelial cell permeability, mesenchymal activations, and fibrosis in diabetic kidneys and interstitial fibrosis in diabetic hearts [17,19]. 

Glomerular fibrosis is one key feature of DKD that is poorly understood [20]. It is characterized by an excess deposition of the extracellular matrix, a loss of capillary networks, and the accumulation of fibrillary collagens, activated myofibroblasts, and inflammatory cells in the glomeruli. Podocyte GR exhibits a crucial role in the regulation of fibrogenic processes in the diabetic glomeruli through regulating Wnt signaling and fatty acid metabolism, thereby affecting the mesenchymal transdifferentiation process in glomerular endothelial cells in diabetic mice, suggesting that GR loss in podocytes disrupts the essential crosstalk between podocytes and endothelial cells [12].

In this Special Issue, Pandey et al. described the implication of various microRNAs and long non-coding RNAs in diabetes and delineated non-coding RNAs (ncRNAs) and their biological networks in diabetes. The authors discussed the clinical trials on diabetes-associated ncRNAs, as well as the functional relevance of the dysregulated ncRNA interactome in diabetes. This knowledge will facilitate the identification of putative biomarkers for the therapeutic management of diabetes and its comorbidities [21].

## 2. New Molecules against Diabetes

The Epidemiology of Diabetes Interventions and Complications study describes how poor initial glycemic control is linked with a higher prevalence of diabetic complications [22]. This phenomenon has been called “metabolic memory.” Controlling glucose levels during only the first year after the diagnosis of diabetes is considerably associated with a future risk of diabetic complications and mortality, even after adjusting glycemic control in the second year after diagnosis [23]. Moreover, hyperglycemia-derived metabolites accumulate abnormally in organs and cause diabetic complications.

In this Special Issue, the authors analyze real-word data from a population-based cohort including 96,643 patients with Type II Diabetes observed for 0.7 million person-years. The authors estimated the risks associated with metformin, and its dose relationship with ESKD, in a propensity-score overlap-weighting cohort using eGFR categories. These data underscore the major benefits and low risk of lactic acidosis with metformin use down to an eGFR of 30 mL/min/1.73 m^2^, and possibly even 15 mL/min/1.73 m^2^, while reinforcing the importance of dose adjustment and the frequent monitoring of eGFR [24]. 

In another study, the authors evaluated the clinical efficacy of continuous subcutaneous insulin infusion (CSII) therapy combined with six classes of oral glucose-lowering drugs (TZDs/metformin/acarbose/GLP-1 receptor agonist/SGLT-2 inhibitor/DPP-4 inhibitor) and carried out their analysis using a network meta-analysis to provide an evidence-based reference for making clinical decisions regarding CSII combined with drugs in the management of type II diabetes (REF). A more outstanding performance was seen with insulin infusion (CSII) combined with metformin, which had the best clinical effect in controlling blood sugar and improving insulin resistance [25].

In this Special Issue, a study by Weber et al. demonstrated the use of the rosiglitazone and its associated increased risk of myocardial infarction. The data suggest that the chronic administration of rosiglitazone does not result in major hidden cardiotoxic effects in myocardial I/R injury models and the inhibition of the antiarrhythmic effects of ischemic preconditioning may have some clinical relevance that needs to be further explored [26].

Cardiovascular disease (CVD) morbidity and mortality are directly associated with diabetes. Individuals with diabetes experience worse clinical outcomes due to heart failure than non-diabetic patients. Hyperglycemia is the main cause that activates the oxidative damage, inflammation, fibrosis, and apoptosis pathways that aggravate diabetic CVD progression. In this Special Issue, Sapian et al. reviewed the phytochemical-based nutraceutical product anthocyanin for diabetic treatment. In preclinical and clinical studies, plants rich in anthocyanin have been reported to attenuate diabetic CVD. The authors unveiled the potential of anthocyanin to be developed as a nutraceutical for therapeutic strategies in the management of CVD associated with diabetes [27].

## 3. Conclusions

It is important to understand the underlying mechanisms of diabetes-induced organ damage. In this Special Issue, we have discussed potent therapeutic regimens for the management of diabetes mellitus, tissue/cell/specific novel biological mechanistic approaches, and clinical data sets. We speculate that this Special Issue will provide basic essential information that could be used in the design of potential therapeutic agents and could help in the management of patients with diabetes mellitus. 
